# Subcellular Localization of Sprouty2 in Human Glioma Cells

**DOI:** 10.3389/fnmol.2019.00073

**Published:** 2019-03-29

**Authors:** Barbara Hausott, Jong-Whi Park, Taras Valovka, Martin Offterdinger, Michael W. Hess, Stephan Geley, Lars Klimaschewski

**Affiliations:** ^1^Department of Anatomy, Histology and Embryology, Division of Neuroanatomy, Medical University Innsbruck, Innsbruck, Austria; ^2^Biocenter, Division of Neurobiochemistry-Biooptics, Medical University Innsbruck, Innsbruck, Austria; ^3^Department of Anatomy, Histology and Embryology, Division of Histology, Medical University Innsbruck, Innsbruck, Austria; ^4^Biocenter, Division of Molecular Pathophysiology, Medical University of Innsbruck, Innsbruck, Austria

**Keywords:** endosome, cytoskeleton, vimentin, ERK, super-resolution, electron microscopy

## Abstract

Sprouty proteins act ubiquitously as signaling integrators and inhibitors of receptor tyrosine kinase (RTK) activated pathways. Among the four Sprouty isoforms, Sprouty2 is a key regulator of growth factor signaling in several neurological disorders. High protein levels correlate with reduced survival of glioma patients. We recently demonstrated that abrogating its function inhibits tumor growth by overstimulation of ERK and induction of DNA replication stress. The important role of Sprouty2 in the proliferation of malignant glioma cells prompted us to investigate its subcellular localization applying super-resolution fluorescence and immunoelectron microscopy. We found that cytoplasmic Sprouty2 is not homogenously distributed but localized to small spots (<100 nm) partly attached to vimentin filaments and co-localized with activated ERK. The protein is associated with early, late and recycling endosomes in response to but also independently of growth factor stimulation. The subcellular localization of Sprouty2 in all areas exhibiting strong RTK activities may reflect a protective response of glioma cells to limit excessive ERK activation and to prevent cellular senescence and apoptosis.

## Introduction

The strength and duration of receptor tyrosine kinase (RTK) activated signaling pathways are tightly regulated by positive and negative regulators. Sprouty2 is the conserved antagonist of the RAS/MAP kinase (ERK) pathway (Mason et al., 2006; Cabrita and Christofori, 2008). The extent and duration of ERK activation are essential determinants in specifying the proliferative and migratory response to growth factors. By sequestering GRB2 from functional signaling complexes and interaction with RAF (Yusoff et al., 2002; Sasaki et al., 2003), Sprouty2 acts as an inhibitor of ERK signaling in various cell types including neurons and glial cells (Hausott and Klimaschewski, 2018). It is stringently controlled at the expression level and by various covalent modifications.

Four different Sprouty isoforms exist which may be co-expressed and bind different proteins in the ERK pathway (Edwin et al., 2009). For example, Sprouty1 and Sprouty4 interact with GRB2 and SOS, respectively, and they combined reduce FGF2-elicited activation of ERK to a greater extent as compared to each individual isoform (Ozaki et al., 2005). Kinases (SRC, MNK1) and phosphatases (PP2A) are required to activate Sprouties while ubiquitination by ubiquitin E3 ligases (c-CBL, SIAH2, NEDD4) and subsequent destruction by the 26S proteasome determines their lifetime (Edwin et al., 2009; Guy et al., 2009). Interestingly, Sprouty2 has been demonstrated to self-assemble into distinct oligomers containing iron and silicon (Wu et al., 2005; Chen et al., 2013).

De-regulation of Sprouty2 is found in a variety of pathological conditions including malignant transformation. Sprouty2 inhibits cell proliferation in response to a number of growth factors, among them EGF, FGF, PDGF and VEGF (Mason et al., 2006). Upon stimulation of RTKs, Sprouty2 translocates from a cytoplasmic pool to the plasma membrane, notably ruffles, where it becomes activated in association with phosphatidylinositol 4,5-bisphosphate (PIP2) and caveolin-1 (Impagnatiello et al., 2001; Yigzaw et al., 2001; Hanafusa et al., 2002; Lim et al., 2002). In line with its role as a negative feedback inhibitor of the ERK pathway, tumor suppressive functions of Sprouty2 have been established in breast, liver, lung and prostate cancers. Sprouty2 gene expression is down-regulated in all of these tumors (Masoumi-Moghaddam et al., 2014). However, in undifferentiated high-grade colon cancer Sprouty2 is elevated and enhances proliferation as well as metastasis (Holgren et al., 2010).

Recently, we corroborated an oncogenic role of Sprouty2 in glial brain tumors (Park et al., 2018). Its expression is up-regulated in highly malignant gliomas which correlates with reduced overall patient survival. Gliomas with high expression of Sprouty isoforms (Sprouty1, -2 and -4) and low expression of NF1 and PTEN are associated with poor prognosis as compared to tumors with a reversed expression pattern (Zhang et al., 2016). Knockdown of Sprouty2 significantly impairs the proliferation of glioma cells *in vitro* and *in vivo*. Interestingly, EGF-induced ERK and AKT activation increase concomitantly resulting in signaling stress with premature S-phase entry. Consistent with these findings, DNA damage response and cytotoxicity are enhanced (Park et al., 2018).

The cell- and context-dependent functions of Sprouty2 are likely related to differences in expression level and/or post-translational modifications which may affect its subcellular localization. Indeed, Sprouty2 function has been shown to be influenced by different levels of interacting proteins such as testicular protein kinase 1 (TESK1). TESK1 overexpression causes Sprouty2 targeting to endosomal membranes but inhibits its translocation to plasma membrane ruffles (Chandramouli et al., 2008). Binding of TESK1 also results in loss of Sprouty2 function as an inhibitor of ERK phosphorylation. Hence, the differential expression of Sprouty2 interacting proteins may result in different spatio-temporal distribution and functionality changes of the protein.

In this study, we investigated the subcellular localization of Sprouty2 in three human glioma cell lines by means of confocal laser scanning, super-resolution stimulated emission depletion (STED), immunoelectron microscopy and immuno-precipitation. We found that Sprouty2 is not homogenously distributed in the cytoplasm but mainly found in small spots. Some of them are attached to vimentin filaments and co-localize activated ERK. We provide evidence that overexpressed Sprouty2 is targeted to all three major endosomal compartments (early, late and recycling) not only in growth factor-stimulated but also in non-stimulated cells. Depending on its expression level, Sprouty2 clearly associates with the plasma membrane as demonstrated before.

## Materials and Methods

### Cells, Plasmids and Reagents

The standard glioma cell lines U251, U87 and SF126 were authenticated by short tandem repeat analysis (Microsynth) and cultured in Dulbecco’s Modified Eagle’s Medium (DMEM, Sigma) or in Roswell Park Memorial Institute (RPMI) medium supplemented with 10% Fetal Bovine Serum (FBS, Gibco), 1% antibiotic-antimycotic (Gibco) and 2 mM L-glutamine (Gibco).

For transient overexpression of Sprouty2, pmNeonGreen-C1-hSprouty2 plasmid was used (mNeonGreen was licensed from Allele Biotech; Shaner et al., 2013). mCherry-Rab5a was a gift from Michael Davidson (Addgene #55126), pLAMP1-mCherry a gift from Amy Palmer (Addgene #45147) and C1-mCherry-Rab11a a gift from Lukas Huber (Biocenter Innsbruck). For shRNA-mediated Sprouty2 depletion, annealed oligonucleotide targeting Sprouty2 (shRNA Target1: GCAGGTACATGTCTTGTCT, shRNA Target2: GATCAGAGCCATCCGAAAC) was inserted into pGLTR-puro plasmid for a stable and conditional RNAi system. For stable transgene expression, lentiviral plasmids encoding wildtype or C-terminally truncated Sprouty2 (FLAG-Sprouty2-CΔ comprising aminoacids 1–253) were cloned by site directed mutagenesis. As previously reported (Park et al., 2018), stable cell lines were established by lentiviral expression. Cells were plated into optical 35 mm μ-dishes (ibiTreat) or on coverslip, serum-starved for 1 h and treated with EGF conjugated to Alexa Fluor-647 (Molecular Probes) for 30 min and fixed.

For transient transfection and subsequent microscopy cells were seeded in cell culture medium and grown until 75% confluency was reached. Twenty-four hours after seeding they were transfected with plasmid DNA using jetPrime™ transfection reagent (Polyplus). The DNA/jetPrime complex was prepared prior to transfection according to the manufacturer’s instructions. For each dish, 2 μg of plasmid DNA was incubated with 6 μl of jetPrime reagent in jetPrime buffer for 10 min and the complex added to the cells. Four hours later the medium was exchanged for normal growth medium. After 24 h the transiently transfected cells were treated and prepared for microscopic examination. All observations are based on cells with medium expression levels of Sprouty2 and endosomal marker proteins.

### Immunostaining

Cells were fixed with 4% buffered formaldehyde solution (made from paraformaldehyde) for 15 min at RT, permeabilized with 0.5% Triton X-100 for 10 min and treated with 10% goat serum for 1 h at RT for blocking unspecific binding sites. Primary antibodies (Abcam: anti-Sprouty2 #60719, 1:160; Cell Signaling: anti-β-tubulin #2128, 1:100; anti-vimentin #5741, 1:100; anti-FLAG #8146, 1:1,600; anti-pERK #9101, 1:400; anti-Rab7 #9367, 1:100) were diluted in 0.3% BSA in phosphate buffered saline (PBS) and incubated overnight at 4°C. After three washings with PBS secondary antibodies (coupled to Alexa Fluor) were added in a dilution of 1:1,000 for 2 h at RT followed by washing and NucBlue/Dapi staining (Molecular Probes). No unspecific binding of the secondary antibodies was observed. At least three independent experiments with *n* > 100 cells were performed.

### Immunoelectron Microscopy

Tokuyasu-cryosection immunogold labeling (Tokuyasu, 1973) of cells overexpressing pmNeonGreen-C1-hSprouty2 was performed according to standard protocols (Liou et al., 1996). Briefly, cells were fixed with 4% (w/v) buffered formaldehyde solution, and thawed ultra-thin cryosections were labeled with mouse anti-Sprouty2 (#60719, Abcam) or rabbit anti-Neongreen (Geley lab) followed by NANOGOLD^®^-Fab’ goat anti-mouse or NANOGOLD^®^-Fab’ goat anti-rabbit IgG (H + L; #2002 and #2004, both from Nanoprobes) visualized by silver enhancement (SE) with HQ-Silver^®^ (#2012, Nanoprobes).

### Immunoprecipitation and Western Blotting

For immunoprecipitation total cell lysates (TCL) of U251 cells stably overexpressing Sprouty2-FLAG were prepared followed by sonication and centrifugation. Dynabeads™ M-280 Sheep anti-mouse IgG (Invitrogen, Carlsbad, CA, USA ) was conjugated with anti-FLAG (Cell signaling #8146, 1:50) overnight at 4°C. Protein lysates were incubated with anti-FLAG-conjugated beads for 1 h at 4°C. After three washes beads were boiled in loading buffer and analyzed together with TCL by immunoblotting (IB). TCL were prepared followed by sonication and boiling. Equal amounts of proteins were separated by sodium dodecyl sulfate-polyacrylamide gel electrophoresis (SDS-PAGE) and transferred to Immobilon-FL-PVDF membrane (Millipore). Membranes were blocked with Odyssey^®^ blocking buffer (LI-COR Biosciences) in PBS and incubated with primary antibodies (Abcam: anti-Sprouty2 #60719, 1:500; Cell Signaling: anti-GAPDH #5174, 1:1,000; anti-tubulin #2128, 1:1,000; anti-vimentin #5741, 1:1,000; anti-FLAG #8146, 1:1,000). The secondary fluorescent-linked antibodies (IRDye^®^ 680RD goat anti-mouse and IRDye^®^ 800 CW goat anti-rabbit, 1:20,000; LI-COR Biosciences) were detected by the Odyssey FC Imaging System (LI-COR Biosciences).

### Imaging and Deconvolution

A confocal laser scanning microscope TCS SP8 (Leica) with a 63× glycerol (N.A. 1.3) objective was used to acquire images with a rate according to the Nyquist-Shannon sampling theorem in a stack of 2 μm thickness (z-interval of 170 nm). Laser intensities were kept constant for comparison of different experiments (excitation and emission wavelengths taken from www.fpbase.org). Confocal images of single cells with medium overexpression levels of Sprouty2 and marker fluorescence were deconvolved using the standard “Deconvolution Express” algorithm in Huygens (version 18.10; SVI, Hilversum, Netherlands). The “Colocalization Analyzer” was then applied to obtain the Object Pearson’s linear correlation coefficient for endosomal Sprouty2 (+1 is total positive linear correlation, 0 is no linear correlation, and −1 is total negative linear correlation). The algorithm searches for colocalizing regions between two channels and calculates the coefficient (*n* > 30, mean ± SD reflecting overlap of Sprouty2 fluorescence with marker fluorescence in 2 μm stacks of single cells). The “optimized search” method was applied for examiner-independent channel thresholding (an extension of the Costes method, but without the assumption that the ideal background combination lies on the regression line). No quantitative co-localization analysis was performed for EGF (glioma cells exhibited highly variable uptake of EGF probably due to different EGFR levels), for vimentin or pERK (both often revealed close association but not true co-localization in high-resolution images).

Super-resolution STED imaging was performed with a STEDYCON (Abberior Instruments) with excitation lasers at 450 nm, 594 nm and 640 nm and a STED laser at 775 nm wavelength (all pulsed). The STEDYCON was mounted at the camera port of an Olympus BX53 upright microscope equipped with a 100× objective (UPLSAPO100 X O/1.4).

## Results and Discussion

**Figure 1:**
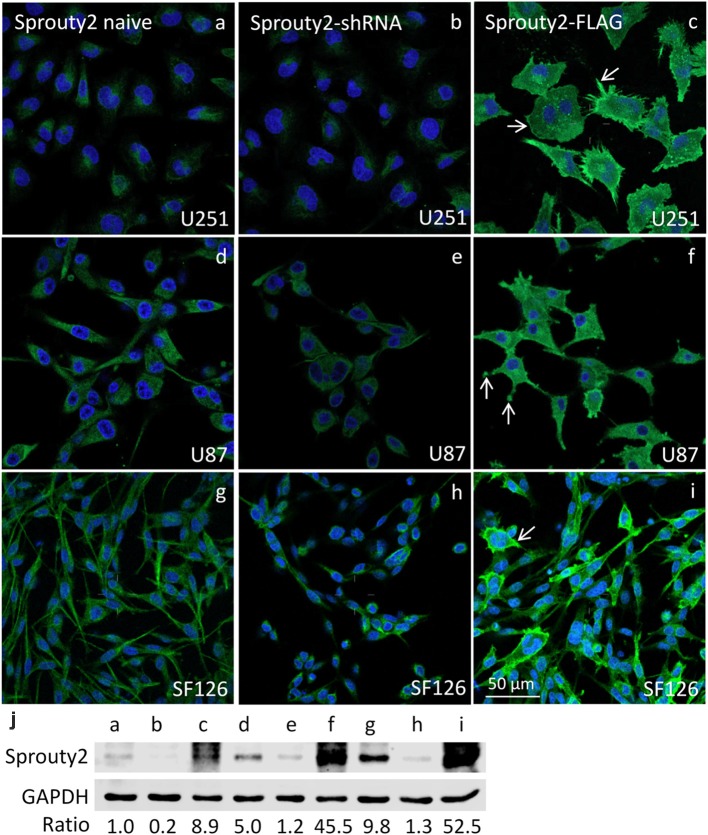
Immunofluorescence against Sprouty2 in glioma cell lines. As compared to naive U251 cells **(a)** stronger labeling is observed in U87 **(d)** and SF126 cells **(g)** Immunostaining of Sprouty2 overexpressing cells reveals prominent labeling of peripheral regions, particularly in cellular protrusions and ruffles (**c,f,i**; arrows). Reduced but not absent labeling is observed in cells with shRNA-mediated Sprouty2 down-regulation **(b,e,h)** which was confirmed by quantitative Western blotting experiments **(j)** demonstrating specific protein bands in naive, shRNA expressing or Sprouty2 overexpressing cells (letters correspond to cells shown in respective images above).

**Figure 2:**
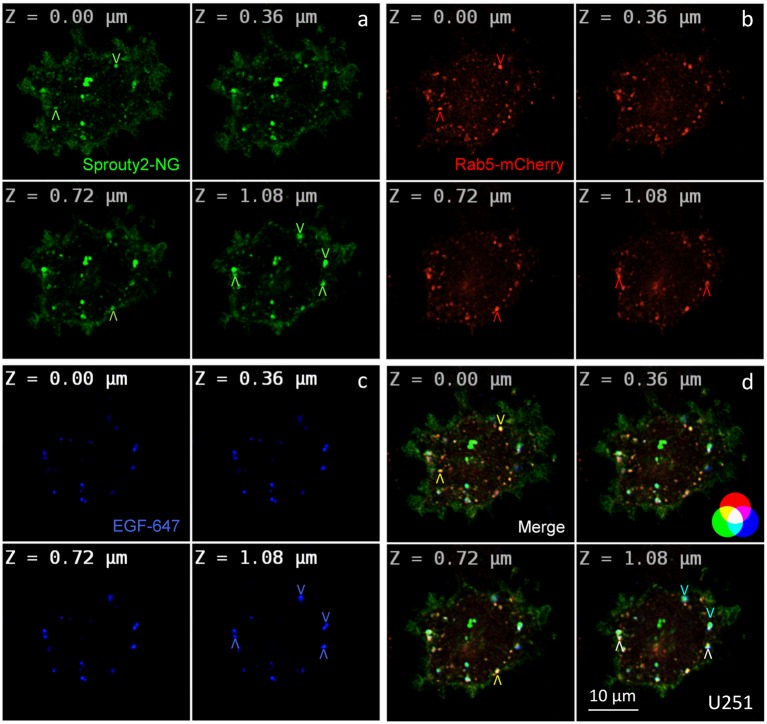
Co-localization of Sprouty2-NeonGreen (Sprouty2-NG, **a**) with early endosomal marker Rab5-mCherry **(b)** at different z-levels of U251 cells 30 min after administration of EGF labeled with Alexa Fluor-647 **(c)**. Merged images reveal predominantly yellow Sprouty2/Rab5, light blue Sprouty2/EGF or white Sprouty2/Rab5/EGF positive endosomes (arrowheads and color scheme in **d**).

**Figure 3:**
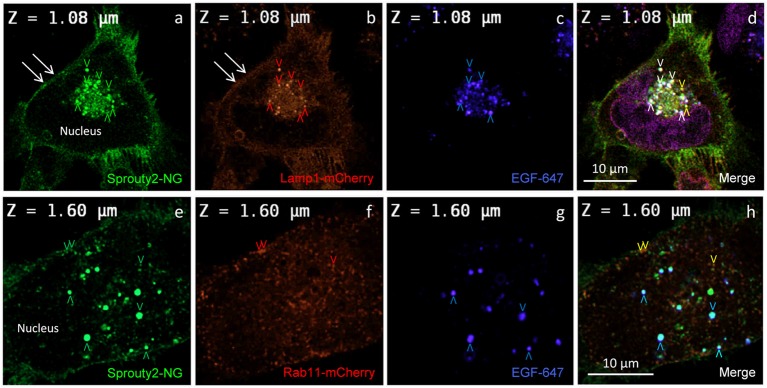
Co-localization of Sprouty2-NG with Lamp1-mCherry **(a–d)** or Rab11-mCherry **(e–h)** in U251 cells treated with EGF-647 **(g)** for 30 min before fixation (arrowheads and color scheme as in Figure 2). Note that Lamp1 labels the plasma membrane which exhibits Sprouty2-NG (arrows in **a,b**) but no nuclear Sprouty2-NG is found **(a,e)**.

The specificity of the Sprouty2 antibody used here was validated in all three investigated cell lines expressing different levels of endogenous Sprouty2 (Figure 1). Endogenous Sprouty2 levels were higher in U87 and SF126 cells than in U251 cells confirming previous biochemical results (Park et al., 2018). Sprouty2 overexpression revealed strong labeling not only in the cytoplasm but also at the plasma membrane and in cellular protrusions (see also Figures 2, 3) which were not visible in non-transfected cells exhibiting endogenous Sprouty2 immunoreactivity. These observations confirm previous studies demonstrating translocation of Sprouty2 from the cytoskeleton to membrane ruffles. The membrane translocation sequences from Sprouty2 are highly conserved and found in the C-terminus which is able to bind PIP2 (Lim et al., 2000). Alternatively, the C-terminal region may undergo palmitoylation or interact with caveolin-1 (Impagnatiello et al., 2001). Moreover, the small GTPase Rac1 induces membrane translocation of Sprouty2 (Lim et al., 2002). Down-regulation of Sprouty2 by specific shRNAs clearly reduced but did not completely abolish Sprouty2 immunostaining and protein levels in lysates of all three cell lines (Figures 1b,e,h,j). The nucleus of glioma cells was devoid of Sprouty2 immunolabeling in contrast to nuclear Sprouty2 found in primary neurons (Hausott et al., 2009).

For overexpression studies, U251 glioma cells were selected because of their low endogenous Sprouty2 levels. Prominent plasma membrane and endosomal localization but no obvious increase in cytoplasmic Sprouty2-mNeonGreen (NG) was observed (Figure 2a). As demonstrated in confocal z-stacks Sprouty2 often co-localized with Rab5 positive early endosomes that mainly accumulated in the cell periphery (Object Pearson Coefficient 0.49 ± 0.08, yellow arrowheads in Figure 2d). Some of them also harbored fluorescently labeled EGF that had been continuously internalized for 30 min. Another subpopulation of Sprouty2 positive compartments contained EGF but not Rab5. Considering the average speed of intracellular EGF trafficking those compartments likely represent late endosomes which, in turn, indicate that Sprouty2 had escaped early endosomal compartments 30 min after EGF uptake (light blue vesicles in merged images of Figure 2d).

In line with these observations, we found that Sprouty2-NG positive vesicles also co-localize endosomal markers such as Lamp1 (Object Pearson Coefficient 0.62 ± 0.1, yellow arrowheads in Figure 3d), Rab7 (Object Pearson Coefficient 0.29 ± 0.1, not shown) and Rab11 (Object Pearson Coefficient 0.28 ± 0.1, yellow arrowheads in Figure 3h). These observations suggest that RTK signaling is influenced by Sprouty2 at every endocytic step until receptors reach the late endosomal or lysosomal compartment. Sprouty2-NG overexpression regularly resulted in binding of Sprouty2 to the plasma membrane labeled by Lamp1-mCherry (Parkinson-Lawrence et al., 2005; Figures 3a,b). Lamp1/Rab7 positive late endosomes were enriched in the perinuclear region, while a plethora of small Rab11 positive recycling endosomes were observed throughout EGF-treated cells (Figure 3f). Most Sprouty2-NG positive late endosomes contained EGF (white arrowheads in Figure 3d), whereas only few EGF positive recycling endosomes associated with Sprouty2-NG were observed (no white or pink spots in Figure 3h).

**Figure 4:**
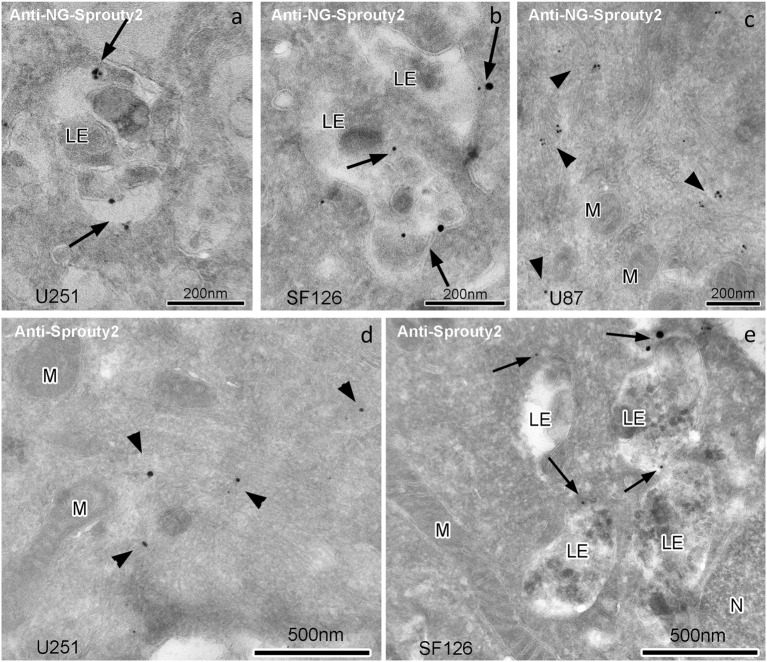
Immunogoldelectron microscopy of U251, SF126 and U87 glioma cells transiently expressing NG-tagged Sprouty2. In U251 **(a)** and SF126 cells **(b)** anti-NG-Sprouty2 label (arrows) is found both at the limiting membrane and inside of late endosomes (in addition to its cytoplasmic distribution), whereas the label in U87 cells (**c**, arrowheads) appears predominantly in the cytoplasm. Anti-Sprouty2 antibodies detect cytoplasmic Sprouty2 in U251 cells **(d)** and at the limiting membrane of late endosomes in SF126 cells **(e)**. LE = late endosome; M = mitochondrion; N = nucleus.

Immunoelectron microscopy applying antibodies against Sprouty2 or NG confirmed that in U251 and SF126 cells endogenous Sprouty2 and overexpressed Sprouty2-NG is localized to the limiting membrane (and inside) of late endosomes in addition to its cytoplasmic distribution (Figure 4). As mentioned above, the binding of Sprouty2 to endosomes may depend on testicular protein kinase 1 (TESK1). TESK1 has been demonstrated to cause targeting of Sprouty2 to endosomal membranes but to inhibit Sprouty2 translocation to plasma membrane ruffles (Chandramouli et al., 2008). On the other hand, Sprouty2 has been demonstrated to interfere with trafficking of activated EGFR at the step of progression from early to late endosomes by interacting with the endocytic regulatory protein *h*epatocyte growth factor-*r*egulated tyrosine kinase *s*ubstrate (HRS or HGS; Kim et al., 2007).

Therefore, the association of Sprouty2 with LAMP1 positive late endosomes/lysosomes does not stop RTK signaling but may be suggestive for prolongation of signaling which may have contributed to the observed increase of ERK activation in tumor cell lines. Thus, Sprouty2 appears to participate in late endosomal signaling complexes in human glioma cells in contrast to murine myoblasts or human pancreatic tumor cells that do not exhibit co-localization of Sprouty2 with Rab7 positive late endosomes (Kim et al., 2007).

**Figure 5:**
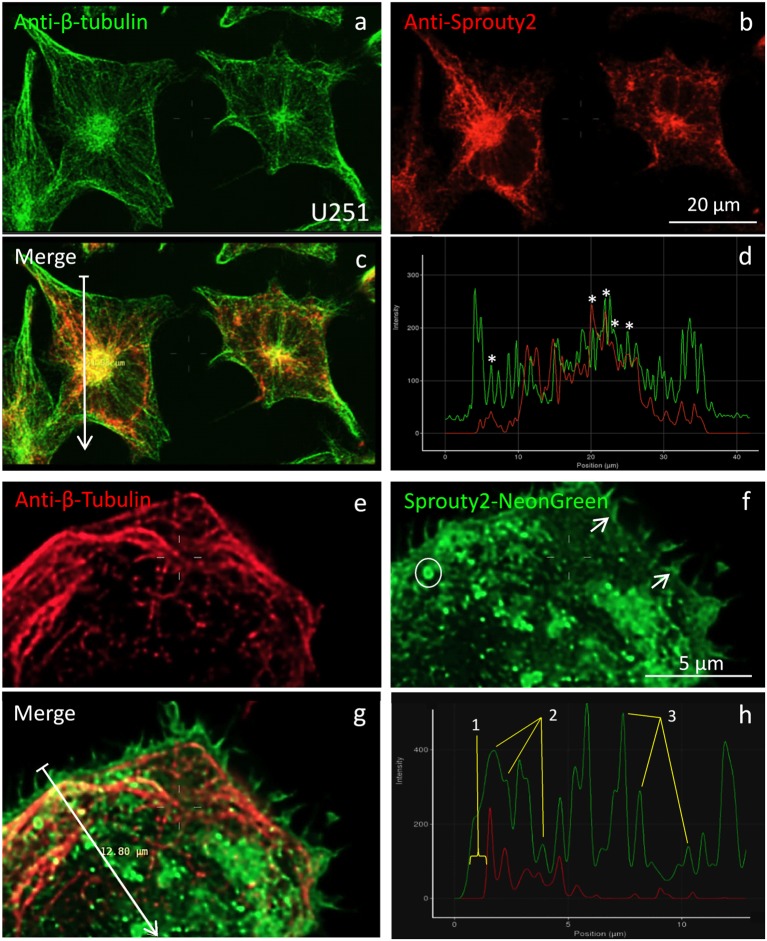
Co-localization of β-tubulin **(a,e**) with endogenous **(b)** or overexpressed Sprouty2-NG **(f)**. Arrows in merged images (arrow length of 41.8 μm in **(c)** and 12.8 μm in **g**) indicate fluorescence intensity distributions along the selected lines **(d,h)**. Stars in **(d)** mark peaks with co-localized immunofluorescence. Numbers in **(h)** correspond to the outer membrane region (1), microtubules (2) and vesicles (3). Note prominent Sprouty2-NG fluorescence at the endosomal (circle in **f**) and at the plasma membrane (arrows in **f**).

According to earlier reports (Lim et al., 2000, 2002; Yigzaw et al., 2001), full-length Sprouty2 may be associated with microtubules. However, we rarely observed overlap or close apposition of endogenous or overexpressed Sprouty2 with β-tubulin in U251 cells (Figure 5). While β-tubulin and endogenous Sprouty2 are both enriched in the perinuclear Golgi area (Figure 5c), the overall distribution of Sprouty2 is markedly different from tubulin in all glioma cell lines tested here. Endogenous Sprouty2 immunolabel was primarily associated with tubulin-negative filamentous structures. Overexpressed Sprouty2-NG was predominantly found in endosomes of variable size and at the plasma membrane (Figures 5f,h).

**Figure 6:**
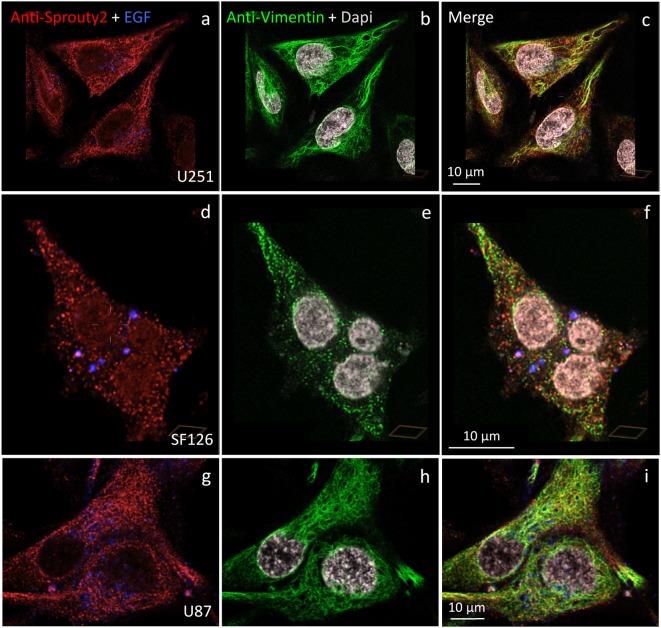
Distinct association of endogenous Sprouty2 with vimentin (intermediate) filaments in U251 **(a–c)**, SF126 **(d–f)** and U87 cells **(g–i)**. Note the punctate staining of Sprouty2 and vimentin in a subpopulation of SF126 cells (treated with EGF-647). However, other SF126 cells (see Figure 7a’), as well as U251 and U87 cells, exhibited the expected filamentous vimentin staining with associated Sprouty2 immunofluorescence (yellow structures in merged images).

**Figure 7:**
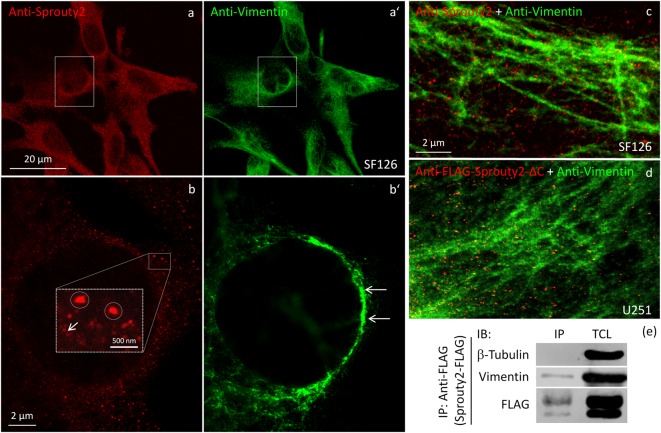
Confocal microscopy of endogenous Sprouty2 **(a)** and vimentin **(a’)** followed by super-resolution (STED) microscopy **(b–d)** reveals endosomal (**b**, inset with higher magnification of spots) and punctate labeling (**b**, arrow) in vimentin rich perinuclear areas of SF126 cells (**b’**). Sprouty2 spots are <50 nm in diameter and partially associated with vimentin filaments **(c)**. Anti-FLAG immunofluorescence against C-terminally truncated FLAG-Sprouty2 demonstrates that Sprouty2 spots (presumably oligomers) form independently of the C-terminus **(d)**. Immunoprecipitation of Sprouty2-FLAG in U251 cells confirms the association of Sprouty2 with vimentin but not with tubulin **(e)**. Anti-FLAG immunoprecipitates (IP) were analyzed in comparison with total cell lysates (TCL) by immunoblotting (IB) probed with antibodies against vimentin, tubulin and FLAG.

We identified the Sprouty2 associated filamentous structures as intermediate filaments in all three glioma cell lines. As shown in Figure 6, the interaction of endogenous Sprouty2 with vimentin filaments was striking. Interestingly, some SF126 cells revealed a punctate pattern of vimentin-positive structures that co-localized with Sprouty2 (Figures 6d–f). Applying super-resolution microscopy the cytoplasmic endogenous Sprouty2 corresponded to large numbers of diffusely distributed small spots (mostly below 100 nm in diameter) with some attached to vimentin filaments or directly co-localizing with vimentin spots (Figures 7a–c). Immunoprecipitation experiments confirmed the binding of Sprouty2 to vimentin (Figure 7e).

**Figure 8:**
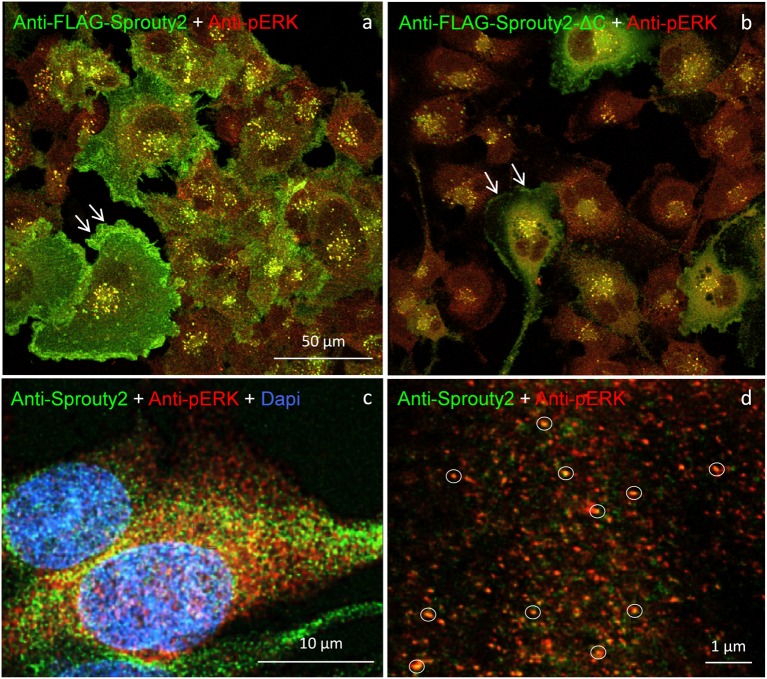
U251 cells overexpressing FLAG-tagged Sprouty2 exhibit immunoreactivity against pERK (yellow spots in **a,b**). High levels of FLAG-Sprouty2 correlate with lower pERK immunofluorescence **(a)**. Note the prominent membrane labeling in cells overexpressing wild-type FLAG-Sprouty2 but reduced fluorescence in FLAG-Sprouty2-ΔC transfected cells (arrows in **a,b**). Endosomes and cytoplasmic Sprouty2 spots partially co-localize pERK in naive U251 cells **(c)**. STED microscopy reveals a large number of fine yellow spots (**d**, circles).

Our observation that Sprouty2 aggregates into 30–50 nm large particles supports the hypothesis of Sprouty oligomerization (Wu et al., 2005; Chen et al., 2013). Sprouty isoforms have been shown to interact with each other to form homo- and heterodimers or oligomers *via* interaction of their Cys-rich sequences (Ozaki et al., 2005). If the C-terminal region (termed Sprouty domain) was purified and investigated by electronmicroscopy, the average particle size was only about 5 nm (Wu et al., 2005). However, we found that the C-terminus of Sprouty2 was not required for the formation of cytoplasmic spots in glioma cells since overexpressed Sprouty2 lacking the Sprouty domain (Sprouty2-CΔ-FLAG) exhibited similar staining patterns as endogenous or overexpressed wildtype Sprouty2 (Figure 7d). However, labeling of the plasma membrane was clearly reduced in Sprouty2-CΔ-FLAG overexpressing cells as compared to full-length Sprouty2 (arrows in Figures 8a,b).

A partial overlap of Sprouty2 with vimentin in the perinuclear region of COS-1 cells has been observed before (Lim et al., 2000). In neurons, vimentin was found to be retrogradely transported as fragments generated by calpain-mediated cleavage (Perlson et al., 2005). The latter study suggested that soluble vimentin acts as a carrier for components of the ERK signaling pathway that are transported to the nucleus in an importin-β dependent manner. These findings prompted us to further investigate a possible co-occurrence of vimentin with Sprouty2 and pERK in human glioma cells. Confirming its canonical role as ERK inhibitor, high Sprouty2 expression correlated with low pERK levels (Figures 8a,b). Nearly all Sprouty2 or Sprouty2-CΔ-positive endosomes and some of the fine cytoplasmic spots (Figures 8c,d) were associated with pERK immunolabel. The latter may reflect the existence of local transportable signaling units which are probably independent of dynein-dependent transport mechanisms along the microtubules since Sprouty2 rarely co-localized with tubulin (Figure 5). The association of Sprouty2 with pERK is expected to be confined to membranes since its binding partners (GRB2 and RAF1) are located mainly at the cell surface or at endosomal membranes. However, GRB2/Sprouty-RAF1-MEK-ERK complexes may bind vimentin to shield the kinase phosphorylation sites, thereby protecting them from PP2A as suggested for neuronal processes (Perlson et al., 2005).

In conclusion, we demonstrate that in human glioma cells cytoplasmic Sprouty2 is mainly localized in small spots attached to vimentin filaments and partially associated with activated ERK. Vesicular Sprouty2 is found in early, late and recycling endosomes. In all glioma cell lines investigated Sprouty2 is found at the plasma membrane only upon overexpression. Elevated Sprouty2 levels are found in highly malignant human glioma and correlate with reduced overall patient survival (Park et al., 2018). The ubiquitous distribution of Sprouty2 in all signaling active subcellular areas may reflect the cellular response to limit excessive ERK activation which may otherwise lead to signaling stress and senescence or apoptosis. Live-cell ERK signaling reporters will be necessary to confirm this hypothesis in imaging experiments. Furthermore, a detailed analysis of expression and subcellular localization of all Sprouty isoforms in human glioma cells is warranted.

## Data Availability

All datasets generated for this study are included in the manuscript.

## Author Contributions

LK: conceptualization. BH, J-WP, TV, MO, MH, SG and LK: methodology and formal analysis. LK: writing—original draft preparation. BH, J-WP, MO, MH, SG and LK: writing—review and editing. BH and LK: funding acquisition.

## Conflict of Interest Statement

The authors declare that the research was conducted in the absence of any commercial or financial relationships that could be construed as a potential conflict of interest.
